# Consolidation cycles, measurable residual disease, and DNMT3A/ASXL1 mutations independently predict relapse-free survival in older adults with acute myeloid leukemia

**DOI:** 10.3389/fonc.2026.1751856

**Published:** 2026-04-15

**Authors:** Xiaolan Wang, Xiaxia Liu, Siying Zang, Jiang Liu, Junyan Zhang, Xialin Zhang, Chunxia Dong, Ruijuan Zhang

**Affiliations:** 1 Department of Hematology, Third Hospital of Shanxi Medical University, Shanxi Bethune Hospital, Shanxi Academy of Medical Sciences, Tongji Shanxi Hospital, Taiyuan, China; 2 Department of Hematology, Linfen Central Hospital, Linfen, China; 3 Department of Clinical Epidemiology and Evidence-Based Medicine, Shanxi Bethune Hospital, Shanxi Academy of Medical Sciences, Taiyuan, China; 4 Department of Hematology, First Hospital of Shanxi Medical University, Research Center for Hemostatic Disorders and Hematologic Malignancies, Shanxi Medical University, Taiyuan, China; 5 Shanxi Academy of Advanced Research and Innovation, Taiyuan, China; 6 Department of Hematology, The Second Hospital of Shanxi Medical University, Taiyuan, China

**Keywords:** acute myeloid leukemia, consolidation therapy, elderly, minimal residual disease, molecular mutations, relapse-free survival

## Abstract

Older adults with acute myeloid leukemia (AML) remain prone to relapse despite achieving remission. We retrospectively analyzed 102 consecutive AML patients ≥ 60 years from two centers in Shanxi, China, who attained complete remission (CR/CRi) after induction with either “3 + 7” or venetoclax plus azacitidine (VEN-AZA). Clinical variables, next-generation sequencing, measurable residual disease (MRD) by multiparameter flow cytometry, and consolidation details were evaluated. Median relapse-free survival (RFS) was 9.7 months (95% CI 7.2-12.2). On multivariable analysis, DNMT3A (HR 2.49, p = 0.005) and ASXL1 (HR 2.88, p = 0.016) mutations and MRD positivity (HR 1.95, p = 0.027) predicted inferior RFS, while receiving ≥ 2 consolidation cycles (HR 0.15, p < 0.001) was protective. The induction regimen was not independently significant. In older AML achieving remission, sustained consolidation and MRD-guided surveillance are pivotal, particularly in patients harboring DNMT3A or ASXL1 mutations.

## Introduction

Older AML patients frequently relapse after achieving complete remission (CR) or CR with incomplete recovery (CRi). Relapse risk is compounded by adverse molecular lesions, comorbidities, and limited tolerance to intensive therapy (1–3). Although “3 + 7” remains the standard induction and venetoclax plus azacitidine (VEN-AZA) has transformed care for unfit patients (4–6), their comparative impact on post-remission relapse-free survival (RFS) and the prognostic influence of measurable residual disease (MRD) and epigenetic mutations in real-world older populations are not well defined.

## Methods

We conducted a two-center retrospective cohort study including adults ≥ 60 years with non-APL AML diagnosed by 2022 WHO criteria. Eligible patients were those aged ≥60 years with newly diagnosed AML (non-APL) according to WHO 2022 criteria, who achieved CR/CRi after induction therapy with either the “3 + 7” regimen or VEN-AZA, had complete follow-up data for relapse and survival, and were treated between January 2018 and December 2023.

Patients with refractory disease, missing critical data, concurrent malignancies, or investigational therapy were excluded. Data were abstracted from electronic records: demographics, comorbidities (Charlson index), gene mutations (53-gene NGS panel), MRD by 10-color flow cytometry (0.1% threshold), induction regimen, and consolidation cycles.

RFS was measured from CR/CRi to hematologic relapse or death; overall survival (OS) from diagnosis. For relapse-free survival (RFS), patients without documented relapse were administratively censored at the date of the last disease assessment. For overall survival (OS), patients alive at the end of follow-up were censored at the date of last confirmed contact. Patients lost to follow-up were censored at the time of last available follow-up. Patients undergoing allogeneic stem cell transplantation were censored at the date of transplantation for both endpoints. Kaplan–Meier and log-rank tests compared survival; Cox models estimated hazard ratios (HR). Two-sided p < 0.05 was significant. Analyses used SPSS v27. Institutional ethics committees approved the study.

## Results

Among 102 patients (median age 68 years), 42 received “3 + 7” and 60 received VEN-AZA (**Table 1**). All patients in the “3 + 7” group received one cycle of induction. In the VEN-AZA group, the median number of induction cycles was 1 (range, 1-2), with the majority (n=55, 91.7%) receiving 1 cycle to achieve CR/CRi. VEN-AZA recipients were older, with higher metabolic comorbidity, and more RUNX1 and IDH2 mutations, while “3 + 7” patients more often underwent intensive consolidation.

**Table 1:** Baseline characteristics of the overall cohort and by induction regimen (3+7 vs. VEN-AZA).

Variable	ALL (n=102)	VEN-AZA (n=60)	3+7 (n=42)	χ^2^ value	p-value
Age (≥75)	11(10.8)	11(18.3)	0 (0.0)	Fisher	P=0.002
Sex (Female)	40(39.2)	21(35.0)	19(45.2)	1.086	P=0.297
CVD	49(48.0)	29(48.3)	20(47.6)	0.005	P=0.943
GD	19(18.6)	11(18.3)	8(19.0)	0.008	P=0.927
MD	38(37.3)	28(46.7)	10(23.8)	5.522	P=0.019
RD	49(48.0)	29(48.3)	20(47.6)	0.005	P=0.943
WBC (≥10 × 10^9/L)	37(36.3)	22(36.7)	15(35.7)	0.010	P=0.922
ANC (≥1.5 ×10^9/L)	39(38.2)	18(30.0)	21(50.0)	4.185	P=0.041
HG (≥70 g/L)	56(54.9)	33(55.0)	23(54.8)	0.001	P=0.981
PLT (≥50 ×10^9/L)	33(32.4)	20(33.3)	13(31.0)	0.064	P=0.800
Blast cell (≥30%)	80(78.4)	49(81.7)	31(73.8)	0.902	P=0.342
ECOG Performance Status (≥2)	89(87.3)	51(85.0)	38(90.5)	Fisher	P=0.551
HCT-CI (≥3)	13(12.7)	10(16.7)	3(7.1)	Fisher	P=0.229
Genetic mutations					
TP53	6(5.9)	5(8.3)	1(2.4)	Fisher	P=0.396
FLT3	19(18.6)	9(15.0)	10(23.8)	1.265	P=0.261
NPM1	20(19.6)	11(18.3)	9(21.4)	0.150	P=0.698
DNMT3A	30(29.4)	18(30.0)	12(28.6)	0.024	P=0.876
RUNX1	14(13.7)	13(21.7)	1(2.4)	Fisher	P=0.007
IDH2	11(10.8)	10(16.7)	1(2.4)	Fisher	P=0.025
WT1	11(10.8)	6(10.0)	5(11.9)	0.093	P=0.760
CEBPA	15(14.7)	9(15.0)	6(14.3)	0.010	P=0.920
ASXL1	10(9.8)	8(13.3)	2(4.8)	Fisher	P=0.191
TET2	11(10.8)	8(13.3)	3(7.1)	Fisher	P=0.518
Number of gene mutations (≥3)	51(50.0)	32(53.3)	19(45.2)	0.648	P=0.421
AML subtype (De novo AML)	97(95.1)	55(91.7)	42(100.0)	Fisher	P=0.076
Prognostic stratification (ELN-2022)				0.005	P=0.943
Favorable and Intermediate	53(52.0)	31(51.7)	22(52.4)		
Adverse	49(48.0)	29(48.3)	20(47.6)		
MFC-MRD	35(34.3)	24(40.0)	11(26.2)	2.090	P=0.148
Consolidation therapy	94(92.2)	55(91.7)	39(92.9)		P=1.000
post-remission regimen switch	32(31.4)	10(16.7)	22(52.4)	14.636	P<0.001
Consolidation therapy cycles (≥2)	69(67.6)	36(60.0)	33(78.6)	3.893	P=0.048
Intensive therapy cycles (≥2)	30(29.4)	6(10.0)	24(57.1)	26.447	P<0.001
Low-intensity therapy cycles (≥2)	44(43.1)	31(51.7)	13(31.0)	4.322	P=0.038
ID/HD-Arac cycles (≥2)	21(20.6)	5(8.3)	16(38.1)	13.385	P<0.001
Antifungal prophylaxis	48(47.1)	33(55.0)	15(35.7)	3.688	P=0.055
Transplantation	10 (9.8)	3(5.0)	7(16.7)		P=0.087

Post-remission regimen switch: Refers to a switch in the post-remission regimen after achieving CR/CRi (e.g., switching between cytarabine/anthracycline-based consolidation and HMA±VEN–based consolidation/maintenance according to tolerance and clinician judgment).

**Figure 1:**
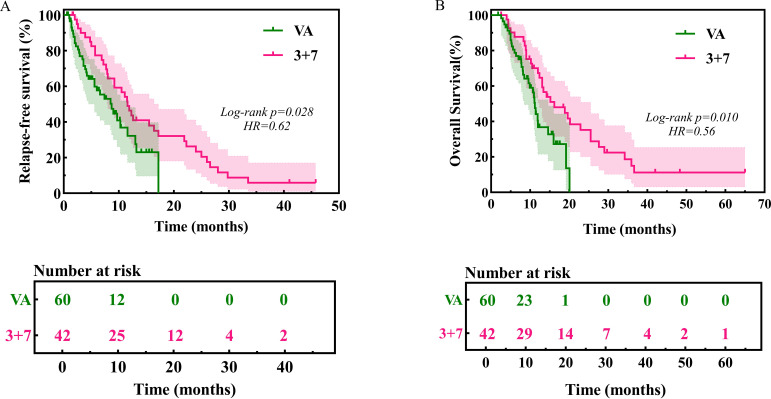
RFS and OS in older adults with AML by induction regimen (3 plus 7 vs venetoclax plus azacitidine). **(A)** RFS from CR or CRi; **(B)** OS from diagnosis. Medians with 95 percent confidence intervals and log-rank P values are reported. The number of patients at risk at selected time points is shown below the survival curves.

At a median follow-up of 15.4 months, 69 (67.6%) relapsed. Median RFS was 9.7 months (95% CI 7.2-12.2); median OS 13.1 months (95% CI 10.7-15.5). Unadjusted analysis favored “3 + 7” over VEN-AZA (RFS 11.6 vs 8.7 months; OS 16.2 vs 11.2 months; p < 0.05; **Figures 1A, B**).

In multivariable models adjusted for age, comorbidities, mutation profile, MRD, and consolidation, induction regimen was not significant. Independent predictors of shorter RFS were DNMT3A mutation (HR 2.49, p = 0.005), ASXL1 mutation (HR 2.88, p = 0.016), and MRD positivity (HR 1.95, p = 0.027). Receiving ≥ 2 consolidation cycles was protective (HR 0.15, p < 0.001) (**Table 2**).

**Table 2:** Univariable and multivariable Cox models for relapse free survival after remission in older adults with AML.

Variable	Univariable analysis		Multivariable analysis	
	HR (95% CI)	p-value	HR (95% CI)	p-value
Age(≥75)	1.50(0.60, 3.77)	0.388		
Sex (Female)	0.86(0.53, 1.41)	0.559		
CVD	0.86(0.53, 1.38)	0.531		
GD	1.22(0.68, 2.17)	0.502		
MD	1.64(0.99, 2.71)	0.056		
RD	1.54(0.96, 2.48)	0.074		
WBC(≥10)	1.16(0.71, 1.90)	0.544		
ANC(≥1.5)	1.19(0.73, 1.91)	0.488		
HG(≥70)	1.37(0.85, 2.21)	0.201		
PLT(≥50)	1.30(0.79, 2.15)	0.305		
Blast cell(≥30%)	1.17(0.65, 2.12)	0.603		
ECOG Performance Status (≥2)	0.40(0.20, 0.79)	0.008		
HCT-CI (≥3)	1.04(0.50, 2.19)	0.914		
Genetic mutations				
TP53	3.25(1.28, 8.22)	0.013	2.75(0.97, 7.79)	0.056
FLT3	1.65(0.92, 2.96)	0.096		
NPM1	1.14(0.59, 2.19)	0.706		
DNMT3A	1.82(1.09, 3.05)	0.023	2.49(1.33, 4.68)	0.005
RUNX1	0.91(0.45, 1.85)	0.799		
IDH2	1.40(0.60, 3.27)	0.442		
WT1	1.07(0.49, 2.36)	0.861		
CEBPA	0.68(0.32, 1.42)	0.298		
ASXL1	2.59(1.21, 5.57)	0.015	2.88(1.22, 6.80)	0.016
TET2	0.90(0.41, 1.97)	0.789		
Number of gene mutations (≥3)	1.45(0.88, 2.39)	0.143		
AML subtype (Secondary AML)	1.75(0.54, 5.66)	0.351		
Prognostic stratification(ELN-2022)(Adverse)	1.45(0.90, 2.33)	0.123		
Induction therapy regimen (3+7)	0.55(0.32, 0.95)	0.030	0.94(0.45, 1.94)	0.862
MFC-MRD	2.05(1.26, 3.32)	0.004	1.95(1.08, 3.54)	0.027
Consolidation therapy	0.35(0.16, 0.77)	0.009	1.52(0.56, 4.16)	0.415
post-remission regimen switch	0.32(0.18, 0.58)	< 0.001	0.55(0.28, 1.11)	0.093
Consolidation therapy cycles(≥2)	0.16(0.09, 0.29)	< 0.001	0.15(0.07, 0.33)	< 0.001
Intensive therapy cycles(≥2)	0.26(0.14, 0.49)	< 0.001	0.72(0.31, 1.67)	0.437
Low-intensity therapy cycles(≥2)	0.42(0.25, 0.69)	< 0.001		
ID/HD-Arac cycles(≥2)	0.53(0.30, 0.95)	0.034	0.90(0.39, 2.04)	0.795
Antifungal prophylaxis	0.76(0.47, 1.23)	0.267		
Transplantation	0.38(0.16, 0.88)	0.025	0.50(0.20, 1.24)	0.133

CVD, cardiovascular disease; GD, gastrointestinal disease; MD, metabolic disease; RD, respiratory disease; WBC, white blood cell; ANC, absolute neutrophil count; HG, hemoglobin; PLT, platelet; ECOG, eastern cooperative oncology group; HCT-CI, hematopoietic cell transplant-composite risk; ELN, european leukemia net; 3+7, intensive induction; MFC-MRD, measurable residual disease by multiparameter flow cytometry; ID/HD-Arac, intermediate/high-dose cytarabine.

## Discussion

These observations are biologically and clinically plausible. It has been reported that DNMT3A mutation potentiates oncogenic drivers, which promotes clonal progression and ultimately enables the persistence or expansion of such clones in relapsed AML (7); ASXL1 mutations remodel chromatin and have been linked to inferior outcomes in AML (8). A recent study reaffirmed that TP53 mutations are strongly associated with disease relapse in elderly AML (9). Patients harboring TP53 mutations exhibit enhanced proliferative capacity and survival of leukemic cells, alongside increased chemoresistance, culminating in a significantly elevated risk of relapse. The lack of a significant prognostic impact of TP53 mutation observed in the present study may be attributable to confounding biases inherent in real-world settings. Measurable residual disease integrates depth of response and clonal fitness and often precedes clinical relapse by weeks to months (10, 11). Its value in predicting outcomes and informing preemptive therapeutic decisions is now well-established across AML subtypes and age groups (12–14). In older patients who are ineligible for allogeneic transplantation, sustained consolidation and MRD informed surveillance are therefore central (15, 16).

In this real-world cohort of older AML patients who achieved CR or CRi, relapse remained frequent, and RFS was therefore selected as the primary endpoint to capture post remission disease control in a population in which intensive salvage and transplantation are often not feasible. OS was analyzed as a key secondary endpoint and showed the same unadjusted direction as RFS. Unadjusted analyses favored 3 plus 7 over VEN-AZA for both RFS and OS, but after adjustment for major prognostic and treatment-related covariates, the induction regimen was not independently associated with RFS. This finding indicates that in our cohort, its impact may be attenuated when considering the number of consolidation cycles, MRD, and molecular profile. However, given the retrospective design and potential selection bias, this finding should be interpreted with caution and does not diminish the established role of induction selection based on patient fitness and disease risk. Prospective validation in larger cohorts is warranted.

The study’s retrospective nature, modest sample size, and heterogeneous consolidation regimens may confound unadjusted comparisons; however, consistency across multivariable analyses and concordance with external data strengthen the conclusions.

In older AML achieving remission, DNMT3A and ASXL1 mutations and MRD positivity independently predict shorter relapse-free survival, whereas the induction regimen does not after adjustment. Receiving ≥ two consolidation cycles is the strongest protective factor. Clinical pathways should prioritize MRD-guided consolidation and close surveillance in patients with DNMT3A/ASXL1 mutations.

## Data Availability

The original contributions presented in the study are included in the article/**Supplementary Material**. Further inquiries can be directed to the corresponding author.
